# A Double-Edged Role for SIRT7 in Cancer: Can Anti-Cancer Immunity Tip the Balance?

**DOI:** 10.3390/ph18121878

**Published:** 2025-12-11

**Authors:** Shahriar Tarighi, Zifan Ning, Andrés Gámez-García, Alejandro Vaquero, Thomas Braun, Alessandro Ianni

**Affiliations:** 1 Department of Cardiac Development and Remodeling, Max-Planck-Institute for Heart and Lung Research, 61231 Bad Nauheim, Germany; shahriar.tarighi@mpi-bn.mpg.de (S.T.); zifan.ning@mpi-bn.mpg.de (Z.N.); 2 Chromatin Biology Laboratory, Josep Carreras Leukaemia Research Institute, Ctra de Can Ruti, Camí de les Escoles s/n, 08916 Badalona, Catalonia, Spain; agamez@carrerasresearch.org (A.G.-G.); avaquero@carrerasresearch.org (A.V.)

**Keywords:** sirtuins, SIRT7, cancer, tumor microenvironment, anti-cancer immunity

## Abstract

**Background/Objectives:** Sirtuin 7 (SIRT7), a nuclear NAD^+^-dependent deacylase, plays multifaceted and sometimes opposing roles in tumorigenesis. By preserving chromatin architecture and genome integrity, SIRT7 protects against malignant transformation; however, once cancer is established, it can either sustain or restrain tumor growth through context-dependent signaling programs, albeit via largely unknown mechanisms. Recent findings have uncovered an additional—and previously underappreciated—dimension: SIRT7’s capacity to modulate anti-cancer immunity. This review revisits the current understanding of SIRT7 in cancer by emphasizing its emerging immunomodulatory functions and influence on the tumor microenvironment. **Methods:** We conducted a comprehensive literature review up to October 2025 using the PubMed database to identify both tumor-intrinsic and tumor-extrinsic mechanisms linking SIRT7 to anti-cancer immunity and to relate the established molecular functions of SIRT7—such as its roles in metabolism, genome maintenance, and inflammatory regulation—to immune regulation. **Results:** SIRT7 directly regulates immune checkpoint expression and T cell metabolic fitness, thereby positioning it as a key node connecting tumor-intrinsic programs with immune surveillance. Moreover, by controlling molecular pathways such as metabolism, genomic stability, and inflammatory responses—both within cancer cells and across other components of the tumor microenvironment—SIRT7 may more broadly influence the immune landscape, orchestrating immune evasion or recognition. **Conclusions:** Deciphering how SIRT7’s tumor-intrinsic and immunomodulatory functions intersect is essential for anticipating the consequences of its pharmacological targeting in cancer. A deeper understanding of this interplay will enable the rational design of combination strategies that integrate SIRT7 modulation with immunotherapy within a precision medicine framework.

## 1. Introduction

The histone deacylase sirtuin 7 (SIRT7) has recently been recognized to play complex, context-dependent roles in tumor initiation and progression by controlling tumor-intrinsic and tumor-extrinsic mechanisms, making it a molecule of growing interest and a promising target for the development of anti-cancer therapies [1].

Cancer remains a leading cause of mortality worldwide, underscoring the ongoing need for more effective and durable therapies [2]. Conventional approaches such as chemotherapy and radiotherapy have provided clinical benefit, but their use is limited by severe side effects and modest long-term efficacy. The advent of precision medicine has ushered in a new era of cancer care, where therapies are increasingly tailored to the molecular features of individual tumors. Genomic profiling of hundreds of cancer-associated genes is now routine in the clinic, enabling the detection of driver mutations, mutational signatures, and structural alterations that inform targeted drug use [3]. In this context, molecules such as SIRT7 are gaining attention as promising candidates for next-generation targeted therapies.

In parallel, our understanding of the complex interplay between cancer cells and their surrounding microenvironment has greatly advanced our knowledge of the intricate mechanisms influencing tumor growth and progression. Tumors are now recognized as complex ecosystems in which malignant cells coexist with diverse non-malignant components—including stromal, vascular, and immune cells—that collectively form the tumor microenvironment (TME). The dynamic crosstalk between cancer cells and the TME profoundly shapes tumor behavior through the exchange of signaling molecules, cytokines, and metabolites, ultimately regulating proliferation, invasion, angiogenesis, and immune evasion [4].

Within this context, anti-cancer immunity has emerged as a central determinant of tumor progression. The immune system continuously monitors tissues to identify and eliminate malignant cells—a process known as immune surveillance. However, tumors can escape immune control by modulating antigen presentation, producing immunosuppressive molecules, or remodeling the TME. These insights have driven the development of immunotherapies, such as immune checkpoint inhibitors, which restore cytotoxic T-cell activity against cancer cells. Despite their success, responses to immunotherapy vary widely, underscoring the need to identify molecular regulators that connect tumor-intrinsic signaling with immune modulation [4].

Moreover, given the central role of the interplay between cancer cells and the immune system, it is not surprising that modern anti-cancer agents have revealed functions extending beyond direct tumor cell killing. Targeted therapies directed against specific signaling pathways can profoundly influence immune responses—either enhancing anti-tumor immunity or suppressing immune surveillance—through both cancer cell-intrinsic and -extrinsic mechanisms. Depending on the molecular and cellular context, such therapies may modulate immunity directly, by altering immune cell function, or indirectly, by reshaping signaling networks within tumor cells and the TME that govern immune activation and suppression. These insights underscore the importance of rationally combining targeted agents with immunomodulatory therapies to harness and amplify immune function for optimal therapeutic efficacy [4,5].

Recent studies have positioned SIRT7 at the crossroads of these processes. SIRT7 has emerged as a multifaceted regulator of tumor initiation and progression, capable of functioning as either a tumor suppressor or a pro-tumorigenic factor depending on the cellular context, through its control of diverse molecular pathways extensively characterized in prior studies [1]. Yet, despite this accumulating evidence, the determinants that govern this dualistic behavior remain elusive—an enigma whose resolution is essential for determining whether SIRT7 can be effectively targeted as a tailored, innovative strategy for cancer therapy. Recently, SIRT7 also emerged as a complex regulator of anti-cancer immunity, indicating a broader involvement of this enzyme in shaping tumor–immune interactions.

This review synthesizes current understanding of the dualistic role of SIRT7 in cancer, with particular emphasis on its newly established functions in orchestrating anti-cancer immunity. In parallel, we integrate evidence on how canonical biological processes governed by SIRT7—including metabolism, genome stability, and inflammatory signaling—intersect with immune regulation and may thereby influence tumor progression. Most critically, we propose that SIRT7-driven immune regulation could represent a pivotal, yet previously underappreciated, mechanistic layer contributing to its context-dependent behavior in cancer, raising the possibility that future mechanistic insights may help determine the therapeutic value of combining SIRT7-targeted interventions with immunomodulatory strategies. To our knowledge, this is the first review to comprehensively propose that immune regulation may serve as a potential driver of SIRT7’s dual role in tumor progression, thereby offering a conceptual framework for future mechanistic studies and translational research.

## 2. SIRT7: A Multifunctional Enzyme Governing Diverse Biological Pathways

SIRT7 belongs to the sirtuin family, a group of seven highly conserved enzymes in mammals (SIRT1–SIRT7). Sirtuins are classified as class III histone deacetylases (HDACs), which, unlike other HDACs, require oxidized nicotinamide adenine dinucleotide (NAD^+^) as an essential coenzyme for their enzymatic activity. They share a highly conserved catalytic domain, while their N-terminal and C-terminal extensions diverge substantially. These variations play critical roles in determining their subcellular localization and mediating interaction with specific targets [6].

Sirtuins display a broad intracellular distribution. SIRT1, SIRT6, and SIRT7 are predominantly nuclear, with SIRT7 mainly localized in the nucleolus. SIRT2 functions primarily as a cytoplasmic enzyme, while SIRT3, SIRT4, and SIRT5 reside within the mitochondria [6,7]. Although distinct sirtuin orthologues share common enzymatic activities and can, in some contexts, act on overlapping protein substrates, they also exhibit distinct substrate preferences, which are often dictated by their subcellular localization. In general, nuclear sirtuins are primarily, but not uniquely, involved in controlling nuclear functions such as chromatin dynamics, epigenetic regulation of gene expression, and DNA repair. Mitochondrial sirtuins participate in metabolic processes, primarily by influencing the activity of mitochondrial enzymes, while SIRT2 regulates different cytoplasmic targets such as components of the cytoskeleton, enzymes, and other proteins [6]. Importantly, sirtuin compartmentalization is dynamic: they are capable of shuttling between cellular compartments in response to internal cues and external stimuli, which enables them to modulate targets beyond their predominant localization, especially under specific physiological or stress-related conditions. Consequently, sirtuins participate in the regulation of a wide array of cellular processes, including metabolism, cellular stress responses, cell proliferation, and survival, and their dysregulation is often associated with numerous human diseases, including cardiovascular, neurological, and metabolic disorders, as well as aging and cancer [7]. Overall, the specific contribution of each sirtuin to these biological pathways is highly dependent on the individual family member, reflecting their distinct regulatory mechanisms and substrate specificities.

For instance, SIRT7 has emerged as a key regulator of numerous biological processes, including metabolism, cellular stress responses, DNA repair, and cell differentiation [7,8,9]. Dysregulation of SIRT7 has been linked to various physiological abnormalities, including impaired regenerative capacity of hematopoietic stem cells (HSCs), inflammatory cardiomyopathy, altered hepatic lipid metabolism, reduced adipogenesis, impaired bone formation and severe osteopenia, delayed hair growth, hearing loss, altered inflammatory responses, and elevated energy expenditure due to thermogenesis, among other effects. Moreover, recent evidence indicates a complex role of SIRT7 in cancer initiation and progression, as discussed below (reviewed in [1,9,10,11]).

**Figure 1:**
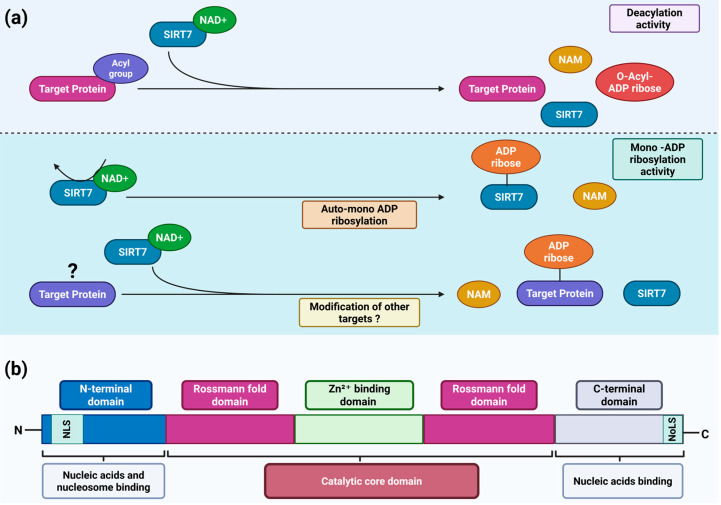
SIRT7 structure and catalytic activities. (**a**) Scheme depicting the main enzymatic activities of SIRT7. SIRT7 is a dual-function enzyme that exhibits both NAD^+^-dependent histone/protein deacylase activity (upper panel) and mono-ADP-ribosyltransferase activity (lower panel); (**b**) Schematic representation of the structural domains of SIRT7 and their corresponding functions. NAD^+^: oxidized nicotinamide adenine dinucleotide; NAM: nicotinamide; NLS: nuclear localization sequence; NoLS: nucleolar localization sequence. See text for details. Created in BioRender. Vazquez Prat, B. (2025) https://BioRender.com/gle6ihc (accessed on 4 December 2025).

SIRT7 orchestrates diverse molecular mechanisms that regulate chromatin dynamics and the epigenetic regulation of gene expression, while extending its activity to non-chromatin targets—including transcription factors, metabolic enzymes, and other components that participate in key signaling pathways [7,8,12]. Although these effects have largely been attributed to the ability of SIRT7 to deacetylate both histone and non-histone proteins, recent findings reveal that it also catalyzes the removal of other acyl groups—such as propionyl, myristoyl, succinyl, and crotonyl—thereby broadening its regulatory repertoire across diverse molecular pathways [1,13,14]. These enzymatic reactions are highly conserved across other sirtuin orthologues [14]. During the deacylation reaction, SIRT7—like other sirtuins—catalyzes the transfer of the acyl group bound to the ε-amino group of a lysine residue within the target protein to the ADP-ribose moiety of NAD^+^, generating O-acyl-ADP-ribose and nicotinamide (NAM) [15]. Beyond its deacylase function, SIRT7 also acts as a mono-ADP-ribosyltransferase—an enzymatic activity shared only with SIRT1, SIRT4 and SIRT6 [14]. In this reaction, the ADP-ribose moiety of NAD^+^ is covalently attached to specific target proteins, concomitantly releasing NAM [14]. Notably, SIRT7 can undergo self–mono-ADP-ribosylation through an autocatalytic mechanism, particularly under conditions of nutrient scarcity. This self-modification is essential for its redistribution to chromatin, where SIRT7 regulates chromatin dynamics and orchestrates the activation of transcriptional programs that enable cellular adaptation to stress. Although additional substrates for this activity have yet to be identified, it is conceivable that SIRT7, through this enzymatic reaction, may regulate broader molecular networks and thereby modulate additional biological functions (Figure 1a) [16].

Human SIRT7 is a protein composed of 400 amino acids. Like all sirtuins, SIRT7 contains a highly conserved catalytic core that includes an NAD^+^-binding site defined by a Rossmann fold and a Zn^2+^-binding domain [14,17]. The three-dimensional organization of this catalytic core forms two adjacent pockets, which are essential for its deacylase and mono-ADP-ribosyltransferase activities, respectively [16]. In addition, SIRT7 possesses distinctive N-terminal and C-terminal domains that set it apart from other sirtuins and critically govern its subcellular localization and functions. As mentioned above, SIRT7 is a nuclear sirtuin which is highly enriched in the nucleolus [7]. This specific localization is mediated by a nuclear localization sequence (NLS; amino acids 61–76) within its N-terminal domain and a nucleolar localization sequence (NoLS; amino acids 392–400) within its C-terminal domain [18]. Moreover, SIRT7 directly binds nucleic acids, an interaction that markedly enhances its catalytic activity. Both the N-terminal and C-terminal domains are required for nucleic acid binding [19,20], with the N-terminal domain recently shown to be essential for nucleosome association and SIRT7-dependent histone deacetylation (Figure 1b) [21,22].

## 3. The Controversial Role of SIRT7 in Cancer

### 3.1. SIRT7 Safeguards Genomic Integrity to Exert Tumor-Suppressive Functions

Since their discovery, sirtuins have emerged as key regulators of cellular homeostasis and genome integrity, acting through diverse molecular mechanisms that enable cells to adapt to metabolic and environmental stressors [23]. Preserving genomic stability—particularly following genotoxic stress—is crucial for preventing the accumulation of deleterious genetic alterations that threaten cellular viability and promote malignant transformation [24]. Consistently, loss of tumor suppressors responsible for preserving genome integrity is tightly associated with increased cancer susceptibility.

Like other sirtuin family members, SIRT7 acts as a critical guardian of genomic integrity. Its depletion increases susceptibility to tumor initiation in mice, under conditions of tumor suppressor loss, carcinogen exposure, or oncogene activation. This is reflected by a higher burden of cancerous lesions and greater tumor size in SIRT7-deficient mice compared with their wild-type littermates [1,25,26,27,28]. This evidence underscores the potent tumor-suppressive role of SIRT7, likely rooted in its ability to stabilize the genome and counteract oncogenic stress. However, no elevated cancer incidence has been observed in SIRT7-deficient mice as compared to controls under physiological conditions, suggesting that loss of SIRT7 is insufficient to initiate tumorigenesis and likely requires additional oncogenic challenges [28].

**Figure 2:**
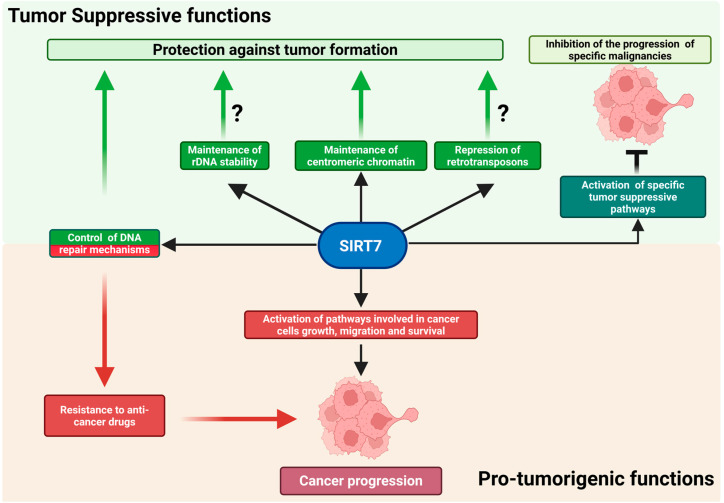
The dual role of SIRT7 in cancer. SIRT7 exerts complex functions in cancer initiation and progression. It acts as a tumor suppressor primarily by maintaining genomic integrity or by activating tumor-suppressive pathways in specific malignancies (upper panel). However, in numerous cancers, SIRT7 acts as a potent pro-oncogenic factor by regulating multiple molecular mechanisms. Further details are provided in the main text. Created in BioRender. Vazquez Prat, B. (2025) https://BioRender.com/krw2j1t (accessed on 4 December 2025).

SIRT7 preserves genomic integrity through multiple, complementary mechanisms, particularly under genotoxic stress. Uniquely localized in the nucleolus, it plays a pivotal role in maintaining the structural and functional integrity of transcriptionally inactive ribosomal DNA (rDNA) repeats that encode ribosomal RNA, thereby contributing to overall genome stability. Because rDNA genes are highly repetitive, maintaining a compact chromatin structure at these loci is essential to prevent aberrant recombination events that can lead to rDNA loss and genomic instability. SIRT7 promotes the establishment of condensed chromatin at rDNA regions by directly deacetylating histones and recruiting heterochromatin-forming factors. Accordingly, SIRT7 depletion leads to rDNA instability, increased cellular senescence, and spontaneous immortalization in vitro (reviewed in [7]; Figure 2).

Although instability of ribosomal DNA has long been recognized as a key contributor to aging in lower eukaryotes such as yeast [7], its contribution to cancer initiation and progression has only begun to emerge. For instance, the rare genetic disorder Bloom syndrome—marked by defective DNA repair and a strong cancer predisposition—is characterized by pronounced instability of rDNA repeats [29]. Consistent with these observations, loss of heterochromatin regulators essential for rDNA maintenance has been associated with rDNA instability, and mutations in these genes are frequently observed in human cancers [30]. Moreover, alterations in rDNA copy number correlate not only with increased tumor susceptibility but also with more aggressive and metastatic cancer phenotypes [31,32]. Thus, it is plausible that SIRT7 exerts its tumor-suppressive functions, at least in part, by safeguarding the integrity of rDNA repeats; however, direct experimental evidence is still lacking (Figure 2) [1,7].

In addition to its role in preserving rDNA integrity, SIRT7 maintains proper chromatin assembly at centromeres by enhancing the activity of histone acetyltransferase 1 (HAT1). Consequently, loss or depletion of SIRT7 disrupts centromeric chromatin organization, promotes aneuploidy, and markedly increases susceptibility to colorectal cancer development in mice carrying predisposing oncogenic mutations [25].

Beyond these functions, SIRT7 further contributes to genomic stability by repressing Long Interspersed Nuclear Element-1 (LINE-1 or L1) retrotransposons through heterochromatin formation at their genomic loci [33,34]. L1 represents the most abundant class of autonomous retrotransposons, accounting for more than 17% of the human genome. They replicate via an RNA intermediate and insert into new genomic locations through a process known as retrotransposition. While transcriptionally active in the germ line and early embryogenesis, L1 elements are normally silenced in somatic cells through epigenetic mechanisms, including DNA methylation and histone modifications. Loss of this repression leads to L1 reactivation, causing insertional mutagenesis and DNA double-strand breaks that promote genomic instability [33,35,36]. Thus, the epigenetic silencing of retrotransposons represents another mechanism through which SIRT7 preserves genome integrity and likely helps counteract tumor initiation (Figure 2).

In addition to these functions, SIRT7 plays a crucial role in orchestrating DNA repair pathways in response to genotoxic stress. Upon the formation of DNA double-strand breaks (DSBs), SIRT7 is rapidly recruited to damage sites, where it deacetylates histone H3 at lysine 18 (H3K18) and desuccinylates histone H3 at lysine 122 (H3K122). These modifications promote local chromatin remodeling, facilitating the recruitment of DNA repair factors and ensuring efficient DSB repair [37,38].

Beyond these direct chromatin-based roles, SIRT7 modulates key components of the DNA damage response (DDR), the signaling network that detects DNA lesions and coordinates downstream repair pathways. The DDR is governed by phosphoinositide 3-kinase–related kinases (PI3KKs)—notably ATM (Ataxia Telangiectasia Mutated), ATR (ATM- and Rad3-Related), and DNA-dependent protein kinase (DNA-PK)—which are activated in a damage-type-specific manner. Activation of these kinases triggers cascades that induce cell-cycle arrest, DNA repair, senescence, or apoptosis, depending on the severity and persistence of the damage, thereby maintaining genomic integrity by promoting accurate repair or eliminating irreparably damaged cells [39,40].

SIRT7 regulates the DDR through several mechanisms. Upon DNA damage, it is recruited to lesion sites, where it promotes ATM deacetylation during the late response stages—an event critical for fine-tuning ATM activity and ensuring proper DNA repair [41]. Paradoxically, ATM can also phosphorylate SIRT7 following anti-cancer drug treatment, inhibiting specific DNA repair pathways and promoting cancer cell survival [42]. These findings indicate that SIRT7 can act both upstream and downstream of ATM, with context-dependent effects on genome stability and drug resistance.

SIRT7 also operates within the ATR signaling axis. Following ultraviolet (UV)-induced genotoxic stress, ATR phosphorylates and activates SIRT7, triggering a downstream signaling cascade that culminates in the stabilization of the tumor suppressor p53. This pathway promotes cell-cycle arrest and the activation of DNA repair programs, thereby contributing to the preservation of genomic integrity [7]. Moreover, activation of SIRT7 by ATR may additionally facilitate its redistribution to chromatin to enhance DNA repair processes, although direct validation of this mechanism is still lacking.

Together, these findings indicate that reduced SIRT7 expression promotes genomic instability, thereby increasing susceptibility to cancer development in response to environmental stressors. Because aging is a well-established risk factor for malignancy—and SIRT7 levels decline across multiple tissues with age—diminished SIRT7 expression may foster a permissive cellular environment for tumor initiation by compromising chromatin integrity and attenuating DNA repair capacity under genotoxic stress (Figure 2) [1,11,34,43].

While the mechanisms outlined above emphasize SIRT7’s critical role in maintaining genome stability, thereby likely preventing malignant transformation, its function in cancer is considerably more complex. Beyond its well-established role in suppressing tumor initiation, evidence suggests that SIRT7 retains tumor-suppressive activity only in a limited subset of malignancies—such as breast cancer—or in tumors characterized by specific genomic alterations, though this remains to be conclusively demonstrated (Table 1). In these contexts, SIRT7 constrains tumor progression by repressing signaling pathways that drive proliferation, survival, epithelial–mesenchymal transition, and metastasis, acting through distinct mechanisms that converge on major downstream cascades, including the TGF-β and AKT pathways and possibly through other mechanisms that remain unknown.

Consistently, reduced SIRT7 levels have been observed in metastatic lesions compared with primary tumors and, in some cases, in malignant cells versus their healthy counterparts. Across these malignancies, lower SIRT7 levels frequently correlate with more dismal prognoses [1,28]. Similarly, reduced SIRT7 expression—along with its association with less favorable clinical outcomes—has also been reported in hematologic malignancies such as acute and chronic myeloid leukemia (AML and CML, respectively) and B-cell acute lymphoblastic leukemia (B-ALL) [1,8,28,47]. Although this evidence suggests a potential tumor-suppressive role for SIRT7 in these cancers, rigorous experimental validation is still lacking, and the mechanisms underlying these putative effects remain to be addressed (Table 1; Figure 2).

Paradoxically, as discussed below, SIRT7 functions as a potent pro-tumorigenic factor in most cancers—even in those where it initially counteracts tumor development. This apparent contradiction reveals a striking duality: while SIRT7 protects against tumor initiation, it can also promote tumor growth and progression once malignancy is established [1].

### 3.2. SIRT7 Is a Prominent Pro-Tumorigenic Factor in Distinct Malignancies

In the vast majority of cancers, including those of the liver, lung, prostate, and thyroid, SIRT7 promotes cancer progression. Supporting this role, SIRT7 is frequently upregulated in these tumors relative to healthy tissues, with elevated expression often correlating with more aggressive phenotypes and poorer clinical outcomes [1].

SIRT7 engages multiple oncogenic mechanisms—such as proliferation, invasion, and metabolic control. These functions are mediated through several molecular pathways (Table 1). These have been thoroughly reviewed elsewhere and are therefore only briefly summarized here [1]. They include the inhibition of tumor suppressors through both epigenetic and post-translational mechanisms, as well as the modulation of key signaling cascades involved in cell growth and survival, notably the mitogen-activated protein kinase (MAPK), protein kinase B (AKT), and tumor protein p53 (TP53) pathways [1,48,49,50].

Furthermore, SIRT7 has emerged as a central regulator of ribosome biogenesis and function. It promotes ribosome production in cancer cells by acting at multiple stages of this process, thereby sustaining the high levels of protein synthesis required for tumor growth. In contrast, SIRT7 can epigenetically repress specific ribosomal protein genes, potentially altering ribosomal composition and translational specificity. Through these complementary activities, SIRT7 may enhance global protein synthesis and fine-tune the translation of oncogenes and tumor suppressors, thereby contributing to cancer progression (Figure 2) [7,48].

Intriguingly, SIRT7 has been increasingly implicated in promoting resistance to anti-cancer therapies that induce DNA damage, positioning it as a potential therapeutic target to overcome drug resistance. Conventional chemo- and radiotherapies eliminate rapidly proliferating tumor cells by inducing extensive DNA damage. Consequently, alterations in DNA repair pathways critically influence therapeutic outcomes: loss-of-function mutations often increase sensitivity, whereas upregulation of repair factors can have the opposite effect [40]. Given SIRT7’s central role in activating DNA repair mechanisms and maintaining genome integrity, it is not surprising that its upregulation enhances cancer cell survival following treatment with DNA-damaging agents. Indeed, accumulating evidence indicates that SIRT7 promotes resistance through the activation or modulation of distinct DNA repair pathways [42,51,52,53,54,55,56,57,58,59]. Moreover, recent evidence shows that, upon treatment with DNA-damaging agents, SIRT7 destabilizes MSH2—an essential component of the mismatch repair (MMR) pathway responsible for sensing and correcting replication errors. This SIRT7-mediated effect may impair recognition of therapy-induced DNA lesions and consequently attenuate downstream cell-death signaling (Figure 2) [42,60].

Altogether, these findings underscore the dual role of SIRT7 in tumor biology, particularly through its control of DNA repair and genomic stability. In non-malignant cells, SIRT7 preserves genome integrity by activating multiple DNA repair pathways and stabilizing chromatin, thereby functioning as a tumor suppressor. In contrast, in cancer cells, SIRT7 can repurpose these same mechanisms and inhibit DNA damage-sensing factors, ultimately fostering drug resistance. Beyond DNA repair, SIRT7 coordinates broader molecular networks that influence tumor behavior, positioning it as an appealing target for therapeutic intervention.

## 4. Toward Therapeutic Targeting of SIRT7 in Cancer

Building on this evidence, the context-dependent functions of SIRT7 make it an attractive, yet challenging and multifaceted therapeutic target. Although no specific SIRT7 activators have been identified, most current efforts focus on developing selective SIRT7 inhibitors, reflecting its predominantly pro-tumorigenic activity in many cancers.

Docking-based screening approaches have identified promising small molecules with high affinity and specificity for SIRT7, while minimizing off-target activity against other sirtuins. Among these, compounds 2800Z and 40569Z were shown to increase the chemosensitivity of hepatocellular carcinoma cells to sorafenib—a clinically approved multi-kinase inhibitor—thereby suppressing tumor progression in mouse xenograft models [61]. Similarly, the SIRT7-targeting compound 97491 was reported to inhibit uterine sarcoma growth in vivo, although its selectivity profile across the sirtuin family remains to be fully characterized (Table 1) [62].

Beyond these synthetic inhibitors, natural compounds have also emerged as a valuable source of SIRT7-targeting molecules. Notably, the bioactive compound YZL-51N, isolated from the cockroach *Periplaneta americana*, was recently identified as a potent and highly selective SIRT7 inhibitor. YZL-51N binds to and occupies the NAD^+^-binding pocket of SIRT7, suppressing its deacetylase function and showing minimal inhibition of other sirtuin family members. In colorectal cancer models, YZL-51N was shown to impair SIRT7-mediated double-strand DNA damage repair following ionizing radiation (IR), thereby enhancing the anti-tumor effect of IR in mouse xenograft models (Table 2) [51].

Finally, the recently discovered cyclic peptide inhibitor lariat 41 exhibits more than fifty-fold selectivity for SIRT7 over other sirtuin isoforms. It markedly increases acetylated lysine 18 on histone H3 (H3K18ac), a principal SIRT7 substrate. Despite these compelling in vitro data, the effect of lariat 41 on cancer progression remains unexplored (Table 2) [63].

However, because SIRT7 promotes tumor progression through mechanisms that extend beyond its enzymatic activity, pharmacological inhibition of its catalytic function alone may be insufficient to achieve durable therapeutic effects. This recognition has led to the formulation of conceptual hypotheses aimed at degrading the SIRT7 protein itself, rather than merely blocking its active site. Among these, small-molecule-induced protein degradation approaches—such as proteolysis-targeting chimeras (PROTACs) and hydrophobic tagging technologies—offer conceptually comprehensive means of suppressing SIRT7. Nevertheless, despite their promise, these SIRT7-directed degradation strategies remain largely hypothetical, and experimental validation is still lacking [49].

Although pharmacological inhibition of SIRT7 shows promise in slowing the progression of various malignancies, the potential adverse consequences of such interventions remain insufficiently characterized. Systemic suppression of SIRT7 could recapitulate phenotypes observed in knockout animal models, including metabolic dysfunctions (e.g., hepatic steatosis), osteopenia, inflammatory disorders, cardiomyopathy, and other abnormalities, as described above [7,8,9]. Therefore, further preclinical and clinical studies are essential to elucidate the safety profile and better define the therapeutic window of SIRT7-targeted approaches.

## 5. Emerging and Potential Roles of SIRT7 in Anti-Cancer Immunity

The immune system maintains tissue homeostasis by detecting and eliminating abnormal cells. Anti-cancer immunity unfolds through a finely orchestrated cascade of events that collectively ensure the recognition and eradication of malignant cells. It begins with the priming, activation, and clonal expansion of tumor-antigen-specific CD8^+^ T cells within draining lymph nodes, followed by their recruitment to the tumor site. Within the TME, these lymphocytes undergo further maturation and functional refinement in specialized immune niches, including tertiary lymphoid structures (TLSs) enriched with B cells, stromal elements, and diverse immune subsets. Once fully activated, cytotoxic T cells execute antigen-specific killing of tumor cells [64].

Malignant cells, however, frequently acquire immune-evasive mechanisms that permit unchecked proliferation and tumor progression. These mechanisms often involve the secretion of immunomodulatory factors—including cytokines, chemokines, and metabolites—that reprogram immune cell behavior and remodel the local microenvironment. Similar processes can also be mediated by non-malignant components of the TME, such as stromal, endothelial, helper T, and myeloid cells, which collectively govern T-cell recruitment, activation, and persistence within tumors [65].

Tumor cells also evade immune surveillance by activating inhibitory signaling pathways, most notably through the upregulation of programmed death-ligand 1 (PD-L1), which directly suppresses cytotoxic T-cell activity. Engagement of PD-L1 with its receptor programmed death 1 (PD-1) on T cells induces functional exhaustion or apoptosis, thereby weakening immune surveillance. Therapeutic blockade of this checkpoint pathway has yielded substantial clinical benefit across multiple malignancies, although therapeutic responses remain heterogeneous and context-dependent, influenced by both tumor-intrinsic and host-related factors [65].

Recent evidence identifies SIRT7 as an emerging regulator of anti-cancer immunity, acting through intricate signaling networks that control not only tumor-intrinsic processes but also the reciprocal crosstalk between cancer cells and the tumor microenvironment, thereby shaping immune surveillance and anti-tumor responses. Several SIRT7-regulated pathways—spanning genomic integrity, inflammatory signaling, and metabolic regulation—are closely intertwined with immune modulation. This interconnected regulatory network suggests that the complex and context-dependent role of SIRT7 in cancer progression may, in part, arise from the combined influence of these processes on the immune–tumor interface, as discussed below.

### 5.1. SIRT7 Modulates Anti-Tumor Immunity Through the Regulation of Immune Checkpoint Expression

SIRT7 has recently emerged as a versatile regulator of anti-cancer immunity, exerting tumor-type-specific effects through distinct molecular mechanisms.

**Figure 3:**
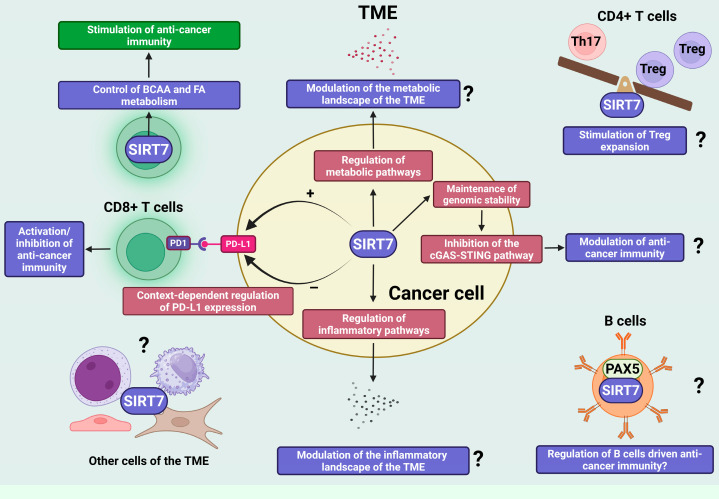
SIRT7 is an emerging regulator of anti-cancer immunity through tumor-intrinsic and -extrinsic mechanisms. SIRT7 is an emerging key regulator of anti-cancer immunity, coordinating both tumor-intrinsic programs and microenvironmental interactions. It modulates immune checkpoint signaling through context-dependent regulation of PD-L1 expression, thereby positively or negatively influencing cytotoxic-T-cell-driven anti-cancer immunity. Beyond this mechanism, SIRT7 shapes immune responses by controlling metabolic pathways within immune and potentially cancer cells. In CD8^+^ T cells, SIRT7 governs branched-chain amino acid (BCAA) and fatty acid (FA) metabolism, supporting metabolic fitness and effector function against tumors. In cancer cells, SIRT7’s central role in the regulation of additional metabolic pathways may collectively remodel the metabolic landscape of the TME and ultimately influence anti-cancer immune responses, though this requires further experimental confirmation. In addition to metabolic control, SIRT7 regulates immune cell function through non-metabolic mechanisms, including modulation of the Th17/Treg balance and regulation of B cell-driven immunity through interaction with lineage-regulating factors such as PAX5—processes that are likely to influence anti-cancer immune responses. Finally, by maintaining genomic stability and restraining activation of the cGAS–STING pathway, SIRT7 links genome protection and inflammatory signaling, thereby potentially influencing anti-cancer immunity. Question marks in the figure indicate mechanisms that are regulated by SIRT7 and are known to influence anti-cancer immunity, although it remains to be determined whether SIRT7 modulates immune responses through these pathways. Created in BioRender. Vazquez Prat, B. (2025) https://BioRender.com/h64j5za (accessed on 4 December 2025).

In hepatocellular carcinoma (HCC), SIRT7 directly deacetylates the transcription factor myocyte enhancer factor 2D (MEF2D), thereby reducing its transcriptional activity and suppressing PD-L1 expression. This downregulation of PD-L1 enhances anti-tumor immune responses [1,46]. These findings highlight the dual role of SIRT7 in HCC: while it promotes cancer cell proliferation, survival, and migration through tumor-intrinsic oncogenic pathways, it simultaneously facilitates immune recognition by diminishing PD-L1-mediated immune evasion. This functional dichotomy provides a strong rationale for combining SIRT7 inhibition with PD-1/PD-L1 immune checkpoint blockade as a potential therapeutic strategy in HCC (Figure 3) [1,46].

In sharp contrast to observations in HCC, evidence from melanoma demonstrates that, under endoplasmic reticulum (ER) stress—a condition frequently experienced by cancer cells—SIRT7 robustly enhances PD-L1 expression by orchestrating alternative stress-responsive transcriptional programs, independent of MEF2A, and linked to activation of the unfolded protein response (UPR), a central adaptive pathway that mitigates ER stress and restores ER homeostasis. Notably, the effect of SIRT7 on PD-L1 expression in melanoma cells under non-stressed conditions is negligible, indicating that, in this malignancy, SIRT7 governs PD-L1 expression through distinct cancer-cell-type-specific regulatory circuits shaped by microenvironmental cues [44].

Additionally, in luminal breast cancer, the positive correlation between SIRT7 expression and the exhaustion marker PD-1 suggests a potential role for SIRT7 in restraining tumor-directed immune responses [66]. Moreover, SIRT7 may also contribute to the upregulation of PD-L1 in pancreatic cancer, but the supporting evidence remains limited, and the molecular mechanisms by which SIRT7 exerts these immunomodulatory effects in both malignancies are not yet delineated [45,66].

Collectively, these observations underscore the tumor-specific nature of SIRT7-mediated immune regulation, particularly in modulating immune checkpoint pathways, which likely operate through distinct molecular programs. These contrasting roles across malignancies may result from tissue- or cancer-specific molecular features acting in concert with contextual cues that reflect the stress state and diverse tumor-intrinsic or extrinsic signals, together reshaping SIRT7-driven transcriptional programs; however, the determinants of this context-dependent behavior remain to be defined.

### 5.2. SIRT7 Shapes Anti-Cancer Immunity by Regulating T Cell Metabolism and Possibly Metabolic Pathways Within Cancer Cells

Immune cells critically rely on balanced metabolic programs to support their survival and to mount effective anti-cancer immune responses. It is well established that alterations in metabolic pathways—either within immune cells or in the tumor microenvironment—can profoundly influence the efficacy of anti-tumor immunity [67].

Cancer cells undergo extensive metabolic reprogramming to meet their energetic and biosynthetic demands, including enhanced glycolysis, altered tricarboxylic acid (TCA) cycle activity, and increased fatty acid oxidation (FAO). Moreover, elevated lipid biosynthesis furnishes essential precursors for membrane formation and generates diverse lipid-derived molecules that support tumor growth and survival. Beyond these, additional metabolic alterations—including those affecting amino acid and nucleotide metabolism—further sustain cancer cell proliferation [68,69]. These adaptations also profoundly reshape the TME, thereby influencing anti-cancer immune responses. For instance, elevated glucose consumption and lactate accumulation in cancer cells limit nutrient availability and suppress cytotoxic T cells activation. Moreover, metabolites derived from the TCA cycle—such as acetyl-CoA, succinate, and α-ketoglutarate—serve as signaling molecules that regulate immune cell differentiation and effector functions. Similarly, lipid metabolic reprogramming can impair anti-tumor immunity by promoting T cell and dendritic cell dysfunction. Therefore, targeting these metabolic pathways has emerged as a promising strategy to restore immune function and enhance immunotherapy efficacy [68].

Increasing evidence indicates that SIRT7 regulates distinct metabolic pathways in both cancer and immune cells, yet the implications of these activities for anti-cancer immunity are only beginning to be elucidated. A recent study showed that SIRT7 inhibits branched-chain amino acid (BCAA) catabolism by desuccinylating key mitochondrial enzymes involved in this pathway. Consistently, depletion of SIRT7 in T cells enhances BCAA catabolic flux, leading to greater utilization of these amino acids and increased generation of acetyl-CoA, which fuels de novo fatty acid biosynthesis. Because T cells rely on tightly controlled levels of these metabolites to support activation and proliferation, loss of SIRT7 results in metabolic dysregulation, impaired expansion and activation, and the promotion of an exhausted phenotype that compromises anti-tumor immunity (Figure 3) [70].

Enhanced BCAA metabolism has also been observed in cancer cells across several malignancies, where such altered metabolic fluxes support tumor growth. Increased BCAA consumption by cancer cells can deplete these essential metabolites within the TME, thereby impairing the metabolic fitness of tumor-infiltrating lymphocytes and suppressing anti-tumor immune responses [71]. It is therefore plausible that SIRT7, similar to its role in T cells, may regulate comparable metabolic pathways through similar molecular mechanisms in cancer cells to modulate BCAA utilization and, consequently, fine-tune the availability of these metabolites in the TME, ultimately influencing anti-cancer immunity. However, this possibility remains highly speculative and requires rigorous experimental validation.

Moreover, the reprogramming of other amino acid metabolic pathways—such as those involving methionine and glutamine—has been recognized as a key driver of cancer progression, while simultaneously shaping anti-tumor immune responses [68,72]. Thus, it is conceivable that SIRT7 may also control additional amino acid metabolic pathways beyond BCAA catabolism, thereby exerting broader effects on cellular metabolism and immune regulation. This hypothesis is supported by findings in lung fibroblasts, where SIRT7 regulates the metabolism of glutamine by repressing the expression of glutaminase 1 (GLS1), a key enzyme that catalyzes the conversion of glutamine to glutamate [73]. Interestingly, loss of GLS1 in breast cancer cells has been found to enhance anti-tumor T cell activation, likely by alleviating competition for glutamine between cancer and immune cells [74]. Further research is warranted to determine whether similar SIRT7-dependent mechanisms operate in cancer cells and thereby influence anti-cancer immune responses.

In addition to these mechanisms, SIRT7 may act as a key regulator of anti-cancer immune responses by controlling lipid metabolism, whose dysregulation in cancer cells leads to fatty acid accumulation within the TME, thereby profoundly influencing immune activity [68]. As discussed above, SIRT7 influences fatty acid biosynthesis by modulating BCAA catabolism in lymphocytes, thereby affecting T-cell activation and anti-tumor immunity [70]. Moreover, SIRT7 exerts a highly context-dependent influence on the regulation of lipid metabolism in both normal and malignant cells. In the liver, SIRT7 appears to exert a protective effect against lipid accumulation, thereby preventing nonalcoholic fatty liver disease (NAFLD) through the modulation of several key signaling pathways, including the attenuation of ER stress and the maintenance of mitochondrial homeostasis. In contrast, other studies have paradoxically reported that loss of SIRT7 can instead protect against high-fat-diet-induced hepatic steatosis by stabilizing TR4/TAK1, a nuclear receptor involved in lipid metabolism and fatty acid uptake (reviewed in [75]). The reasons behind these contrasting effects remain highly debated and have largely been attributed to the different mouse models and genetic backgrounds used across studies. Nonetheless, further research is needed to clarify the context-specific influence of SIRT7 on hepatic lipid metabolism in vivo [75]. Furthermore, in liver cancer cells, SIRT7 promotes the biosynthesis of cholesterol, fatty acids, and triglycerides through activation of the methyltransferase PRMT5, which methylates the transcription factor SREBP1a, thereby triggering a SREBP1a-dependent transcriptional program that drives the expression of lipogenic and cholesterogenic genes [76]. Collectively, these findings highlight a markedly context-dependent role for SIRT7 in lipid metabolism that may differ between normal and transformed cells. Although further research is warranted, it is plausible that SIRT7 influences anti-cancer immunity, at least in part, through the regulation of lipid metabolic pathways in specific cellular contexts.

Finally, SIRT7 also regulates other metabolic pathways that may influence anti-cancer immunity. For instance, SIRT7 suppresses glycolysis by deacetylating and inhibiting the activity of the key glycolytic enzyme phosphoglycerate kinase 1 (PGK1), as well as by lowering the levels of hypoxia-inducible factors (HIFs) [12,77,78]. Given that enhanced glycolysis in cancer cells suppresses anti-tumor immunity through metabolic competition with T cells, it is plausible that SIRT7 upregulation in certain malignancies may indirectly promote immune responses by limiting glycolytic flux. However, this hypothesis remains to be experimentally validated.

Overall, these findings suggest that while SIRT7 supports anti-tumor immunity—partly by regulating metabolic programs in T cells—it may also reshape the tumor microenvironment through distinct metabolic networks operating in cancer cells, especially when its expression is deregulated. Clarifying these mechanisms remains an important goal for future research.

### 5.3. Non-Metabolic Regulation of Immune Cell Function by SIRT7: Potential Implications for Anti-Cancer Immunity

Besides its role in controlling metabolism and activating cytotoxic T cells, SIRT7 may also influence anti-cancer immunity by regulating additional immune subsets, particularly CD4^+^ T cells. These cells are pivotal orchestrators of anti-tumor responses, shaping the activity of CD8^+^ T cells, natural killer cells, B cells, macrophages, and antigen-presenting cells, primarily through the secretion of subset-specific cytokines. Moreover, under certain conditions, CD4^+^ T cells can directly mediate tumor cell elimination [79]. Recent evidence indicates that SIRT7 profoundly affects the functional balance between T helper 17 (Th17) cells and regulatory T (Treg) cells—two major CD4^+^ T-cell subsets with opposing immunological roles [80]. Tregs help maintain immune equilibrium by releasing anti-inflammatory cytokines, whereas Th17 cells secrete IL-17 and drive pro-inflammatory responses. Alterations in the Th17/Treg ratio exert context-dependent effects on tumor progression, either promoting or restraining cancer growth depending on the tumor microenvironment [81]. Mechanistically, SIRT7 promotes Treg expansion while limiting Th17 differentiation by desuccinylating and thereby inhibiting the transcription factor STAT3, which in turn reduces the expression of its downstream target genes [80]. Given the critical role of the Th17/Treg axis in tumor immunity, these findings suggest that SIRT7 may indirectly modulate anti-cancer immune responses through this pathway. Nonetheless, the relevance of this regulatory axis in cancer remains to be clarified and should be carefully considered when assessing the therapeutic potential of SIRT7 inhibitors.

Recent evidence also indicates that SIRT7 plays a significant role in B-cell differentiation by directly deacetylating the transcription factor paired box 5 (Pax5), a master regulator of B-cell lineage commitment [8]. Tumor-infiltrating B cells are emerging as key players in cancer immunity, capable of exerting both pro- and anti-tumor effects through diverse mechanisms—including cytokine secretion, modulation of CD8^+^ T cell function, and production of tumor-specific antibodies—depending on the tumor context [82]. Notably, Pax5 also contributes to B-cell activation and is expressed in tumor-infiltrating B cells within the TME, suggesting functional relevance in shaping anti-cancer immunity [83,84]. Although its role in B cell-mediated tumor immunity remains insufficiently defined, the SIRT7–Pax5 axis may represent a possible mechanism of B cell regulation within the TME, with important implications for anti-cancer immunity. Further experimental validation will be essential to substantiate this hypothesis and clarify its relevance in SIRT7-dependent immune regulation (Figure 3).

### 5.4. SIRT7 May Link Genomic Stability and Inflammation to the Regulation of Anti-Cancer Immunity

SIRT7 may also influence anti-cancer immunity through its central role in maintaining genomic integrity, thereby positioning it as a potential mediator at the intersection between genome stability and immune surveillance.

Cancer is characterized by pronounced genomic instability, manifested by the progressive accumulation of point mutations and chromosomal aberrations during tumor evolution. These genetic alterations can lead to the expression of aberrant proteins that are recognized by the immune system as non-self-antigens or neoantigens, thereby eliciting immune responses that can counteract tumor growth [85]. In parallel, genomic instability can engage signaling pathways with strong immunomodulatory potential that further shape anti-tumor immunity.

A central example is the cyclic GMP–AMP synthase–stimulator of interferon genes (cGAS–STING) pathway, which originally evolved to detect cytosolic DNA from invading pathogens and to induce type I interferon production together with NF-κB-dependent inflammatory programs that promote pathogen clearance. More recently, this pathway has been shown to sense self-derived double-stranded DNA that accumulates in the cytoplasm as a consequence of genomic instability or as reverse-transcribed DNA generated by reactivated endogenous retrotransposons. This activation triggers innate immune signaling, modulating inflammatory responses during aging, and potentiating anti-cancer immune surveillance [86,87,88,89]. Consistent with these findings, pharmacological strategies targeting the DNA damage response can enhance tumor susceptibility to immunotherapy, at least in part by activating the cGAS–STING axis [90]. Moreover, cancers characterized by elevated genomic instability—such as those deficient in mismatch repair (MMR)—display increased responsiveness to immune checkpoint blockade [60,91].

Given the established role of SIRT7 in maintaining genomic stability and repressing retrotransposon expression, it is not surprising that a functional connection between SIRT7 and the cGAS–STING pathway has been identified. Inhibition of SIRT7 in human mesenchymal stem cells stimulates the innate immune signaling through activation of the cGAS–STING pathway, driven by derepression of LINE-1 (L1) retrotransposons—an event associated with increased cellular senescence [34]. Likewise, pharmacological inhibition of SIRT7 activates the cGAS–STING pathway in hepatic stellate cells (HSCs), whose activation is a key pathological event in liver fibrosis. This activation promotes HSCs senescence and enhances natural killer (NK)-cell-mediated immune clearance of these cells, thereby exerting a protective effect against hepatic fibrogenesis [92].

SIRT7 may also prevent activation of this pathway by limiting the accumulation of cytoplasmic double-stranded DNA—both through promoting DNA-repair mechanisms and by preventing the release of rDNA fragments generated by homologous recombination, through chromatin stabilization [7]. Despite clear evidence that SIRT7 inhibits the cGAS–STING pathway across multiple cellular contexts, the relevance of this process to anti-cancer immunity remains largely unexplored. If similar mechanisms operate in tumor cells, SIRT7 could attenuate immune surveillance by restraining pathway activation, thereby limiting anti-tumor responses. Elucidating this connection will be essential to define the contribution of SIRT7-dependent genome protection to cancer immunobiology (Figure 3).

Finally, beyond its potential involvement in the cGAS–STING axis, SIRT7 may also influence anti-cancer immunity through additional signaling cascades that regulate inflammatory processes. Notably, SIRT7 has been reported to exert both stimulatory and inhibitory effects on inflammation, depending on the cellular context—in malignant as well as non-malignant cells—by controlling distinct molecular mechanisms [1,11,93,94,95,96,97,98,99]. Thus, deregulated SIRT7 activity in cancer cells could profoundly reshape the inflammatory and immunological landscape of tumors, ultimately influencing anti-cancer immunity (Figure 3).

## 6. Discussion and Future Directions

SIRT7 has emerged as a pivotal regulator of tumorigenesis whose influence extends from genome maintenance to metabolic control and immune modulation. SIRT7 counteracts malignant transformation; yet once this process has taken place, it preserves tumor-suppressive functions only in a restricted subset of malignancies, while in the majority of tumors, it adopts tumor-supportive roles. These opposite functions are governed by complex signaling cascades that either restrain or sustain tumor progression, respectively [1,7].

The contrasting effects of SIRT7 across malignancies may reflect differences in their mutational landscapes, signaling architectures, and cellular contexts. Oncogenic mutations may alter pathways that influence SIRT7 expression or activity, likely affecting the enzyme through post-translational modifications, altering its subcellular localization, or modifying its interactions with specific binding partners, thereby reshaping its downstream actions. In parallel, tumor-type-specific repertoires of cofactors and chromatin regulators could fine-tune SIRT7 function, producing tissue-restricted outcomes from shared molecular circuits. Although this model is plausible, the mechanisms through which context-dependent molecular features modulate SIRT7 activity—and dictate its divergent roles across cancer types—remain largely unresolved.

Emerging evidence indicates that SIRT7 performs intricate, multilayered functions in anti-cancer immunity by modulating key pathways, particularly those governing immune checkpoint expression and T cell metabolism [1,44,45,46,70]. Moreover, SIRT7 shapes several molecular networks that are intimately connected to the regulation of anti-tumor immunity and may contribute to the broader orchestration of immune responses against cancer. However, this hypothesis remains largely inferential and demands rigorous experimental substantiation. This speculative nature therefore represents a central limitation of this review.

Although accumulating evidence implicates SIRT7 in immune regulation, it remains unclear whether its contrasting effects on tumor progression arise from distinct functions in cancer cells versus the immune compartment. For instance, as discussed above, SIRT7 appears to play a dual role in liver cancer, acting as a prominent pro-tumorigenic factor by modulating cell-intrinsic mechanisms while simultaneously stimulating anti-cancer immunity [46]. Comparable dual behavior may occur in other malignancies, where variations in SIRT7 expression and tumor-intrinsic features collectively shape the tumor microenvironment and influence anti-tumor immune responses. Clarifying how these layers intersect—and to what extent SIRT7’s differential roles are driven by its effects within tumor cells versus immune populations—remains a central challenge. Progress in disentangling these regulatory layers has been limited by the constraints of current experimental models.

Most studies have relied on xenograft systems in which cancer cells, genetically modified to alter SIRT7 expression, are implanted into immunocompromised mice [1]. While these models have yielded valuable mechanistic insights into the tumor-intrinsic functions of SIRT7 and, to a limited extent, its interactions with the tumor microenvironment, they inherently fail to capture the contribution of immune cells and the complex, dynamic crosstalk between tumor and immune compartments. Moreover, most in vivo studies to date have utilized constitutive, global SIRT7 knockout mouse models [1]. Although these models have been instrumental in uncovering broad roles for SIRT7 in cancer biology, they may obscure cell-type-specific functions—particularly those distinguishing tumor cells from components of the TME, including immune populations. To overcome these limitations, future research should systematically compare the effects of SIRT7 modulation on tumor progression in immunodeficient versus immunocompetent hosts, using syngeneic and/or allograft models that better reflect the complex interplay between cancer cells and the immune system. In parallel, experimental approaches that enable selective inactivation of SIRT7 in tumor cells within transgenic mouse models—while preserving its activity in the surrounding microenvironment—such as dual recombinase systems that couple SIRT7 deletion with oncogene activation, hold considerable promise for elucidating the cell-type-specific roles of SIRT7 in tumorigenesis. Likewise, targeted manipulation of SIRT7 in immune cells or other components of the TME could further clarify its diverse and context-dependent roles in both tumor progression and immune regulation.

Disentangling the relative contributions of SIRT7 to cancer-intrinsic and immune-mediated mechanisms will be essential for defining its role in tumorigenesis and for determining whether the tumor microenvironment can tip the balance of its impact on cancer progression. These investigations will further clarify whether pharmacological manipulation of SIRT7 activity through systemically administered compounds could exert complex immunomodulatory effects—acting not only on cancer cells but also within the TME—to either enhance or restrain anti-cancer immunity. Such insights would be instrumental in determining whether combining SIRT7-targeting agents with immunomodulatory approaches could improve therapeutic efficacy, particularly in specific malignancies, and could ultimately prove crucial for the development of precision-tailored therapies centered on SIRT7.

## 7. Conclusions

SIRT7 integrates genomic maintenance, oncogenic signaling, and immune regulation into a unified network that shapes cancer progression. Rather than functioning as a simple tumor suppressor or promoter, SIRT7 acts as a context-dependent molecular rheostat, balancing cellular resilience with immune recognition. Understanding how these molecular circuits are rewired in different tumor settings will be crucial for defining when and how SIRT7 becomes an ally or an adversary in cancer. Future research should not only clarify the cell-type-specific functions of SIRT7 but also determine whether its modulation can be exploited to fine-tune the interplay between tumor-intrinsic programs and anti-cancer immunity, ultimately guiding the design of next-generation therapeutic strategies.

## Figures and Tables

**Table 1:** Main roles of SIRT7 in distinct malignancies. The table summarizes the pro-oncogenic and tumor-suppressive roles of SIRT7 in various malignancies.

Role of SIRT7	Function	Malignancies
Pro-oncogenic factor	Stimulation of proliferation, migration and survival of cancer cells.	Liver, lung cancer, thyroid cancer, prostate cancer, pancreatic cancer, colorectal cancer, melanoma, sarcoma, endometrial and ovarian cancer [1].
	Inhibition of anti-cancer immune responses.	Melanoma and pancreatic cancer [44,45].
Tumor suppressor	Prevention of tumor formation following exposure to carcinogens or activation of oncogenes/tumor suppressor loss.	Skin cancer, liver cancer, colorectal cancer [1,25,26,27].
	Stimulation of anti-cancer immune responses.	Liver cancer [46].
	Inhibition of tumor growth and migration.	Breast cancer, oral squamous cell carcinoma [1].
	Other possible functions?	Reduced SIRT7 levels are associated with worse clinical outcomes in AML, CML and B-ALL suggesting potential tumor-suppressive roles [8,47].

AML: acute myeloid leukemia; CML: chronic myeloid leukemia; B-ALL: B-cell Acute Lymphoblastic Leukemia.

**Table 2:** SIRT7 inhibitors and their anti-cancer activities. The table summarizes the main SIRT7-targeting compounds, including their chemical structures, half-maximal inhibitory concentration (IC_50_) values and documented anti-cancer effects. Chemical structures for all inhibitors—except Lariat 41—were drawn using Marvin (Demo Version 25.3.51, ChemAxon). The structure of Lariat 41 was reproduced in Microsoft PowerPoint based on its published chemical structure [63].

Name	Anti-Cancer Activity
2800Z (IC_50_: 134–165 µM) 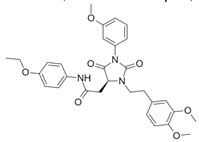	Enhances the chemosensitivity of liver cancer cells to sorafenib and suppresses tumor growth in mouse xenografts by blocking SIRT7-mediated inhibition of apoptosis [61].
40569Z (IC_50_: 13 µM–96 µM) 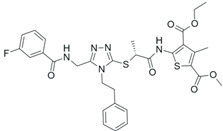	Enhances the chemosensitivity of liver cancer cells to sorafenib and suppresses tumor growth in mouse xenografts by blocking SIRT7-mediated inhibition of apoptosis [61].
97491 (IC_50_: 0.325 µM) 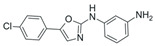	Inhibits uterine sarcoma progression in mouse xenograft models by blocking SIRT7-mediated inhibition of apoptosis [62].
YZL-51N (IC_50_: 12.7 µM) 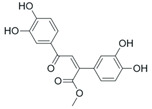	Attenuates SIRT7-mediated DNA repair, enhancing the sensitivity of colorectal cancer cells to DNA-damaging agents [51].
Lariat 41 (IC_50_: 2.7 µM) 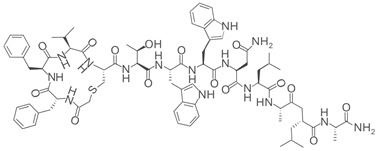	Increases histone acetylation via SIRT7 inhibition. Anti-cancer activity not assessed [63].

## Data Availability

Not applicable.
